# Adjuvant treatment with Capecitabine in patients who received orthotopic liver transplantation with incidental diagnosis of intrahepatic cholangiocarcinoma. Implications on DPYD polymorphisms assessment: report of two cases and review of the literature

**DOI:** 10.1007/s00280-025-04756-x

**Published:** 2025-03-12

**Authors:** Carolina Liguori, Simona Magi, Alessandra Mandolesi, Andrea Agostini, Gianluca Svegliati-Baroni, Andrea Benedetti Cacciaguerra, Alessandro Parisi, Elisa Tiberi, Marco Vivarelli, Andrea Giovagnoni, Gaia Goteri, Pasqualina Castaldo, Rossana Berardi, Riccardo Giampieri

**Affiliations:** 1 Medical Oncology, Department of Clinical and Molecular Sciences, University Politecnica delle Marche, Ancona, 60126 Italy; 2 Department of Biomedical Sciences and Public Health, Section of Pharmacology, University Politecnica delle Marche, Ancona, 60126 Italy; 3 Services Department, Laboratory of Pharmacogenomics (Hospital Hygiene Unit), University Hospital “Azienda Ospedaliero Universitaria delle Marche”, Ancona, 60126 Italy; 4 Anatomic Pathology Unit, University Hospital “Azienda Ospedaliero Universitario delle Marche”, Ancona, 60126 Italy; 5 Department of Radiological Sciences, Division of Clinical Radiology, University Hospital “Azienda Ospedaliero Universitaria delle Marche”, Ancona, 60126 Italy; 6 Department of Clinical, Special and Dental Sciences, University Politecnica delle Marche, Ancona, 60126 Italy; 7 Liver Injury and Transplant Unit, University Politecnica delle Marche - University Hospital “Azienda Ospedaliero Universitaria delle Marche”, Ancona, 60126 Italy; 8 Hepatobiliary and Abdominal Transplant Surgery, Department of Experimental and Clinical Medicine, University Politecnica delle Marche - University Hospital “Azienda Ospedaliero Universitaria delle Marche”, Ancona, 60126 Italy; 9 Department of Oncology, University Hospital “Azienda Ospedaliero Universitaria delle Marche”, Ancona, 60126 Italy; 10 Anatomic Pathology Unit, Department of Biomedical Science and Public Health, University Politecnica delle Marche, Ancona, 60126 Italy

**Keywords:** Dihydropyrimidine dehydrogenase, Fluoropyrimidine, Orthotopic liver transplant, cholangiocarcinoma, Case report

## Abstract

In recent years, assessing dihydropyrimidine dehydrogenase (DPD) activity has become crucial for cancer patients undergoing 5-fluorouracil (5FU)-based chemotherapy due to the life-threatening toxicity associated with reduced DPD function. The methods for evaluating DPD activity have evolved, with the analysis of DPYD polymorphisms in blood samples becoming the preferred approach. As the indications for liver transplantation are increasing—particularly due to a rise in cases of cholangiocarcinoma (CCA) and non-resectable colorectal liver metastasis—more cancer patients with a history of liver transplantation may experience disease relapse. Furthermore, 5-fluorouracil chemotherapy is a standard treatment for both cancers. This growing need to evaluate DPD activity in transplanted livers arises because standard tests conducted on blood samples reflect the activity of native liver tissue and may produce misleading results. This paper presents two clinical cases from 2022 to 2023 involving patients who underwent successful liver transplants but were later diagnosed with intrahepatic CCA in the explanted liver. Both patients were subsequently prescribed capecitabine as adjuvant chemotherapy, making it essential to assess DPD activity in donor liver tissue to ensure safe treatment protocols. However, there are currently no established guidelines for this specific patient group. If we follow standard clinical practice, this critical analysis will be insufficient, as it only describes the DPD activity of the native liver. It is imperative to determine the DPD activity of the transplanted liver. In summary, this case report highlights the importance of managing this complex situation effectively.

**Supplementary Information:**

The online version contains supplementary material available at 10.1007/s00280-025-04756-x.

## Intro

Over the past decade, the assessment of dihydropyrimidine dehydrogenase (DPD) activity has become standard of care for all cancer patients who are candidate to receive 5-fluorouracil (5-FU)-based chemotherapy combinations [1–3]. Indeed, the majority of DPD-related fluoropyrimidine metabolism occurs in the liver [4]. A deficiency in DPD activity is associated with life-threatening toxicity due to prolonged drug exposure [5].

Several methods have been assessed to detect DPD deficiency, including phenotyping —either direct or indirect measurement of enzyme activity—or genotyping, which involves the detection of inactivating polymorphisms in the *DPYD* gene.

Blood uracil testing is the most widely used method for indirectly measuring enzyme activity in the absence of genotyping. It operates on the assumption that DPD converts physiologic uracil (U) into dihydrouracil (UH2). The assessment of the U/UH2 ratio or, alternatively, the measurement of plasma uracil levels, offers a relatively inexpensive and rapid means of determining DPD activity. Pre-treatment uracil concentrations greater than 16 ng/ml have been reported to be associated with an increased risk of side effects related to fluoropyrimidine-related side [6]. In contrast, direct measurement of DPD activity is instead performed by evaluation of peripheral mononuclear cell DPD activity (PBMC-DPD activity) [7]. Indeed, a correlation between PBMC-DPD activity and DPD liver enzymatic activity has been demonstrated [8]. Nevertheless, indirect phenotyping approaches are more frequently employed, owing to a higher number of clinical applications and research relative to direct approaches [9].

Another form of assessment is based on *DPYD* polymorphisms analysis [10–13]: several relatively common polymorphisms have been described for *DPYD* gene. This genotyping process identifies pathogenic polymorphisms in the *DPYD* gene, primarily c.1905 + 1G > A (rs3918290), c.1679T > G (rs55886062), c.2846 A > T (rs67376798), and c.1236G > A (rs56038477). In the event of a heterozygous polymorphism being present, it is recommended that the fluoropyrimidine dose be reduced by either 25% or 50%, depending on the specific type of polymorphism and the consequent reduction in enzyme activity. For patients who are homozygous carriers of the c.1236AA variant, a 50% dose reduction has been shown to significantly reduce the risk of toxic complications. Conversely, in instances of other homozygous polymorphisms, the use of fluoropyrimidines is contraindicated, owing to the heightened risk of complications [14]. Evaluation of *DPYD* polymorphisms in patients’ blood samples is increasingly recognized as a reliable method of DPD activity evaluation among medical oncologists. This test is based on the premise that *DPYD* polymorphisms that could be identified in DNA derived from circulating blood cells should mirror *DPYD* polymorphisms in liver cells. Consequently, it enables the prediction of increased toxicity risk during 5FU-based chemotherapy [15].

Recent advances in cancer therapy may influence these forms of DPD testing. In particular, heterologous liver transplantation (LT) has been increasingly investigated as a potential treatment for various cancer types with predominant liver involvement [16–20]. In this patient group, *DPYD* polymorphisms analysis on peripheral blood samples may not accurately predict the risk of life-threatening toxicity with 5-FU. These tests would reliably assess the DPD activity of the explanted liver, rather than the transplanted liver.

Phenotypic testing based on plasma uracil levels would be less reliable for assessing DPD activity in this patient group. Elevated levels of alanine aminotransaminase (ALT), aspartate aminotransaminase (AST) and creatinine are frequently detected in these patients and have been linked to higher plasma uracil, even with normal DPD activity [21].

Furthermore, it should be emphasized that liver transplantation is increasingly employed for various tumor types. Among these, several— such as colorectal cancer or cholangiocarcinoma— where 5-FU remains the mainstay of treatment [19, 20, 22, 23].

Given these factors, the number of cancer patients who have undergone liver transplantation and may require DPD activity assessment to receive 5-FU based chemotherapy is expected to increase. However, there is limited guidance on how to manage these instances. This paper aims to suggest an alternative to standard *DPYD* polymorphism analysis to accurately assess the risk of toxicity in this group of patients.

In this study, we present the clinical cases of two patients admitted to our hospital in 2022–2023 and who underwent liver transplantation. Post-transplant, both patients were diagnosed with intrahepatic cholangiocarcinoma in the explanted liver. Consequently, they were advised to commence capecitabine adjuvant chemotherapy [24]. *DPYD* polymorphisms assessment was performed on the donor’s liver tissue in both patients to evaluate DPD activity in the transplanted liver.

## Patients

Patient A, was a 62 years old male patient at the time of diagnosis. He underwent combined liver-kidney transplantation in December 2021 for decompensated alcohol-related liver cirrhosis. Prior-transplantation chest-thoracic computed tomography (CT) scan also showed the presence of a single nodule in the IVA liver segment that was suggested to be primary liver cancer rather than metastasis (Supplementary Fig. 1).

The histologic report in the explanted liver confirmed the presence of pT2 pN0 well-differentiated mass-forming cholangiocarcinoma, with perineural and lymphovascular invasion. Staging was performed by chest-abdomen CT scan that proved to be negative for metastases. Adjuvant chemotherapy with Capecitabine monotherapy (1250 mg/sqm twice daily dd1-14 once every 3 weeks for 8 cycles) was advised, in order to reduce risk of disease relapse.

As there was availability of donor’s liver histological tissue, *DPYD* polymorphism assessment was performed in the liver tissue rather than in the circulating blood of the patient.

Tumor DNA was extracted from tissue; fixed in formalin and embedded in paraffin, with MagCore automatic extractor with MagCore Total DNA FFPE One-Step Kit (Diatech Pharmacogenetics). *DPYD* polymorphisms assessment was performed in the liver-derived DNA. The following 4 polymorphisms were analyzed: c.1905 + 1G > A (rs3918290), c.1679T > G (rs55886062), c.2846 A > T (rs67376798) and c.1129–5923 C > G (rs75017182). DPYD genotyping was carried out by using the CE-IVD and European Molecular Quality Network (EMQN) certified qRT-PCR EasyPGX^®^ ready DPYD kit (cod. RT026, Diatech Pharmacogenetics, Jesi, IT) [25].

Using different oligo mixes, the kit allows the detection of the *DPYD* gene polymorphisms by allelic discrimination, resulting in the co-amplification of both the mutant sequence (labeled with FAM) and/or the wild-type sequence (labeled with HEX). The run was performed on the Easy PGX qPCR instrument (Diatech Pharmacogenetics, Jesi, IT).

**Fig. 1:**
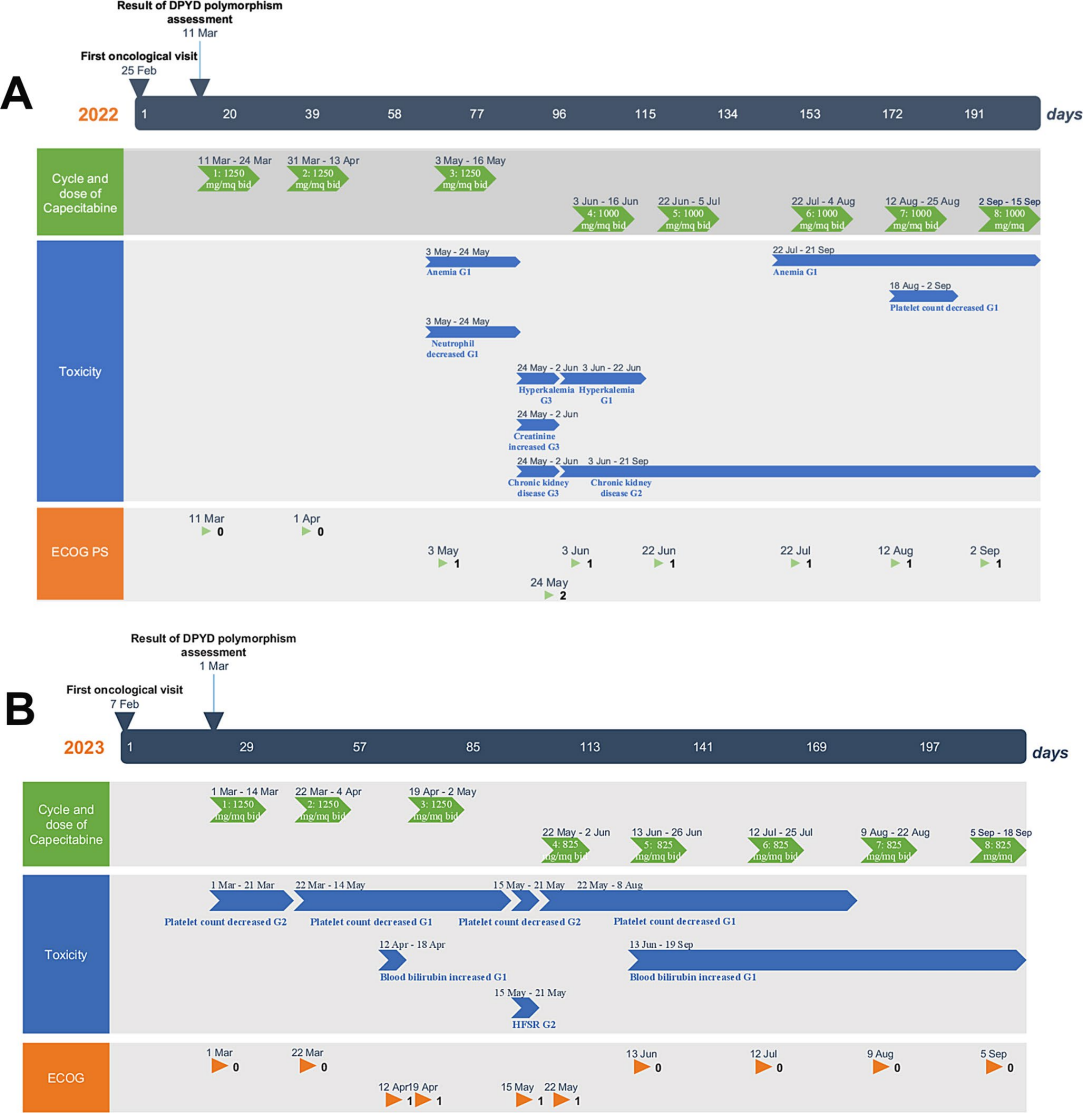
**A**: Patient A timeline. **B**: PATIENT B timeline

*DPYD* polymorphisms analysis was available 16 days after the initial oncology visit.The analysis showed that no polymorphisms of DPYD gene were present in the donor’s liver. He started in March 2022 treatment with Capecitabine as previously planned. At baseline, the patient presented with mild renal impairment, which permitted the initiation of Capecitabine-based adjuvant chemotherapy without the need for dose reduction. Patient A timeline (Fig. 1A) summarises the toxicity profile and treatment schedule of Capecitabine treatment. As it can be seen, all 8 cycles of treatment with Capecitabine were successfully completed, with no signs of severe toxicity (defined as greater than Grade 3 toxicity graded by National Cancer Institute – Common Terminology Criteria for Adverse Events, NCI CTCAE ver 6.0). Hematological toxicity was manageable: patients experienced as worse hematological toxicity G1 anemia and G1 thrombocytopenia. As non-hematological toxicities, patient only experienced one isolated episode of increased creatinine (G3 NCI CTCAE) that led to temporary delay of the 4th cycle of chemotherapy with Capecitabine Due to moderate renal impairment (eGFR 45 ml/min), the dosage was reduced from 1250 mg/sqm twice daily to 1000 mg/sqm twice daily. In September 2022 he concluded adjuvant treatment with Capecitabine and is currently in active follow-up with no signs of disease relapse.

Patient B was a 68 years old male patient at the time of initial tumor diagnosis. He was diagnosed with liver cirrhosis in March 2021. He was also diagnosed with Barcelona Clinic Liver Cancer Stage B hepatocellular carcinoma (Supplementary Fig. 3) and in December 2022 he underwent orthotopic liver transplantation after having received trans-arterial chemoembolization twice. In the explanted liver, the pathologist confirmed that one of the nodules was a well-differentiated hepatocellular carcinoma (stage pT2 pN0) but it was found also found that the other nodule was a well-differentiated intrahepatic cholangiocarcinoma (stage pT1 pN0).

After proper staging with chest-abdomen CT scan that excluded distant metastases, patient was advised to start adjuvant treatment with Capecitabine monotherapy (1250 mg/sqm twice daily dd1-14 once every 3 weeks for 8 cycles) because of the concomitant presence of resected intrahepatic cholangiocarcinoma. At baseline, Patient B had a low platelet count (G2 NCI CTCAE) due to splenomegaly, which does not require a dose reduction.

Due to availability of liver tissue from the donor, we were able to perform *DPYD* assessment on liver tissue, following the same procedure that was described before. 21 days after the initial request for *DPYD* testing, patient resulted to be wild-type for *DPYD* polymorphisms and thus started of Capecitabine monotherapy on March 2023.Patient B timeline (Fig. 1B) summarises schedule of treatment and treatment compliance and toxicity. As it can be seen, after 2 cycles of treatment, dose of Capecitabine had to be reduced down to 825 mg/sqm twice daily instead of 1250 mg/sqm due to low platelet count. Patient experienced also palmar-plantar-erythrodysesthesia syndrome (G2 NCI CTCAE). Patient concluded his adjuvant treatment on October 2023, after having received 8 cycles of treatment. He is currently in follow-up with no actual signs of disease relapse. Patient’s timeline can be found in Fig. 1B.

## Discussion

We have presented two clinical cases of patients who have received post-operative 5-FU based chemotherapy after having received liver transplantation. There is limited accumulated knowledge concerning differences in toxicity profile of this group of patients compared with those who did not receive liver transplantation.

5FU-based chemotherapy is a mainstay of treatment for patients with either colorectal, gastric, pancreatic and biliary tract cancer, both in the palliative and adjuvant setting [26–29]. It has been extensively proven in past studies how 5FU metabolism is mainly mediated by the liver: indeed, less than 1% of administered 5-FU is turned into active metabolites whereas the remaining portion of the drug is excreted by the kidneys and more than 80% of it is metabolized in the liver [4].

In most recent trials and everyday clinical practice, Capecitabine has been used as substitute to 5-FU: there are some differences in terms of toxicity compared to standard 5-FU but it has been proven that Capecitabine metabolism is similar to standard 5-FU metabolism once that the oral prodrug has been converted into its active metabolites leading to 5-FU accumulation in the tumor [30, 31].

Several guidelines have included DPD deficiency screening as means to reduce the risk of life-threatening 5FU-based toxicity.

For example, in a previously published study of Laures et al. [32] plasma uracil concentration was able to identify patients with DPD deficiency and who would have higher risk of 5-FU related toxicity: median toxicity score was higher in the unscreened group vs. those patients who underwent DPD activity assessment for each cycle of chemotherapy (0 vs. 1; *p* < 0.001). Cumulative toxicity score during course of treatment was also higher in the unscreened group (0 [min 0; max 4] vs. 0 [min 0; max 6]; *p* = 0.0028). This difference in toxicity could be mainly traced to different doses of 5-FU that were administered to patients based on DPD assessment as there was a statistically significant difference in 5-FU dose reductions in patients with DPD-deficiency (1056 ± 351 mg/m^2^/week vs. 1233 ± 251 mg/m^2^/week in non-deficient patients *p* < 0.001).

In another study of Meulendijks [6], baseline high uracil concentration (greater than 16 ng/ml) rather than increased uracil/dihydrouracil concentrations were associated with global severe toxicity (OR 5.3, *P* = 0.009), severe gastrointestinal toxicity (OR 33.7, *P* < 0.0001), toxicity-related hospitalisation (OR 16.9, *P* < 0.0001), as well as fatal treatment-related toxicity (OR 44.8, *P* = 0.001).

In addition to direct and indirect measurement of DPD enzyme activity, another way to assess DPD activity is based, as we have already stated before, on *DPYD* polymorphisms analysis:

In a large prospective dutch trial [14], 1181 patients were screened for DPD activity by assessment of DPYD polymorphisms analysis in peripheral blood cells (genotypic approach). Eighty-five patients (8%) were found to be heterozygous *DPYD* allele variant carriers. Toxicity was higher in patients who were DPYD allele variant carriers (33 [39%] of 85 patients) compared to wild type patients (231 [23%] of 1018 patients; *p* = 0·0013). In addition to that, 5FU dosing strategy based on *DPYD* allele variant genotype was able to reduce the risk of 5FU related toxicity: in c.1905 + 1G > A allele carriers the relative risk of toxicity was 1.31 (95% CI 0.63–2.73) when comparing different dosing strategy based on the genotype vs. historical controls. Other alleles were also associated with differences in relative risk of toxicity whenever different dosing strategy based on genotype was not used (no toxicity vs. 4.30 [2.10–8.80] in c.1679T > G carriers, 2.00 [1.19–3.34] compared with 3.11 [2.25–4.28] for c.2846 A > T carriers, and 1.69 [1.18–2.42] compared with 1.72 [1.22–2.42] for c.1236G > A carriers). Authors concluded that for c.1905 + 1G > A and c.1679T > G carriers, a 50% initial dose reduction was adequate whereas for patients with c.1236G > A and c.2846 A > T carriers, it should be investigated a dose reduction greater than 25% that is commonly suggested.

In 2020, the European Medicines Agency (EMA) recommended both methods for pre-treatment DPD deficiency testing in clinical practice: phenotyping using endogenous uracil concentration or genotyping for *DPYD* risk variant alleles [1]. In Italy, the AIOM-SIF (Associazione Italiana Oncologia Medica – Società Italiana di Farmacologia) Working Group recommends the *DPYD* genotype test be performed on germinal DNA extracted from peripheral blood to identify DPD deficiency [33]. The mutations under analysis are c.1236G > A (rs56038477), c.1679T > G (rs55886062), c.1905 + 1G > A (rs3918290), c.2194G > A (rs1801160), and c.2846 A > T (rs67376798).

All the studies that have been cited before have included a rather heterogeneous group of patients who were candidate to receive 5-fluorouracil based chemotherapy: however, they can offer only limited insight on what should be done in a patient who received liver transplantation.

As previously stated, *DPYD* genotyping is able to accurately describe DPD liver activity, as it has been shown to have good correlation between *DPYD* assessment in peripheral blood cells and DPD activity in the liver [8]. However, *DPYD* genotyping would not be informative for DPD liver activity in a patient who underwent liver transplantation, as the genotype of peripheral blood cells would reflect the genotype of the explanted liver, rather than the donor’s liver.

Based on these assumptions, phenotypic approach might then be considered as the best alternative to genotypic approach as plasma uracil levels would reflect fluoropyrimidine metabolism of the transplanted liver similarly to patients who have never received liver transplantation.

There are, however, a few factors that might reduce the usefulness of phenotypic approaches for DPD assessment in liver transplanted patients; in particular, indirect methods based on plasma uracil methods might have less reliable results due to the frequent occurrence of confounders:

Decreased estimated glomerular filtration rate (eGFR) lower or equal to 45 ml/min was associated to an increase of plasma uracil concentrations leading to an increase of falsely positive results of DPD deficiency assessment when performed with this method [34].

Indeed, patients with gastrointestinal cancer have impaired renal function due to pre-existing conditions of loss of fluids, thus reducing the accuracy of plasma uracil concentration as form of DPD activity assessment: a large retrospective French study [21] showed that 12.7% of Caucasians had uracilemia greater than 16 ng/mL, suggesting that the phenotypic approach might overestimate the diagnosis of DPD deficiency.

In addition to that, there were also warnings concerning significant differences in measured pretreatment uracil levels, most likely as a result of pre-analytical factors in a large multicenter retrospective analysis [35].

Moreover, looking specifically at patients who received orthotopic liver transplantation, it has been described that in the 50 days following the procedure as much as 25% patients experience an increase in serum creatinine levels [36]. This fact might have a relevant impact on the reliability of plasma uracil testing as way to assess DPD activity, particularly in the time-frame when post-operative adjuvant treatment should be started.

Looking at these data, from a hypothetical point of view, there is a lack of evidence concerning the reliability of standard ways to assess DPD activity, either by phenotyping or genotyping approach, in patients who have received liver transplantation. Indeed, the data concerning not only DPD assessment, but also available evidence on the side effects of 5FU based treatment in patients who underwent liver transplantation is also limited:

Brandi et al. [37] reported the outcomes of a series of patients who underwent liver transplantation for liver metastases from colorectal cancer. In the three cases that were reported, even after having experienced disease relapse thus leading to retreatment with 5FU based chemotherapy combinations, toxicities that were recorded were manageable. There was a general increase in the risk of developing severe chemotherapy related side-effects: one patient experienced G4 neutropenia after having received chemotherapy with FOLFOXIRI post-transplant whereas the most severe toxicity that could be seen before liver transplant was G2 neutropenia; one other patient also experienced G3 neutropenia post-transplant when receiving FOLFOX-based chemotherapy whereas in the pre-transplant setting the most severe toxicity that was recorded was G2 neutropenia.

Lin et al. [38] have published a meta-analysis of adjuvant systemic therapy after liver transplantation for HCC: authors report that adjuvant chemotherapy appears to be well-tolerated. The studies included in the meta-analysis are rather heterogeneous and have also included patients who have received doxorubicin and/or cisplatin based chemotherapy rather than 5FU-based treatment. Description of adverse events was also of anecdotal nature, thus reducing its usefulness in describing accurately the likelihood of toxicity.

In addition to that, prospective or retrospective data from patients with liver metastases who have received chemotherapy after liver transplantation are lacking. For example, in previous clinical trials of liver transplantation in patients with liver metastases from colorectal cancer such as SECA-1 and 2 trials [39, 40] no adjuvant chemotherapy was given after liver transplantation thus limiting our knowledge concerning risk of 5FU toxicity in patients who received liver transplantation.

Finally, the recently published TRANSMET trial [16] reported the outcomes for patients with unresectable liver metastases from colorectal cancer who, after having received chemotherapy, were randomised to continue chemotherapy with or without liver transplantation. The study showed that there was an improvement in overall survival for patients who received liver transplantation against those who just continued chemotherapy (HR:0.37, 95%CI:0.21–0.65, *p* = 0.0003). However, only 46% patients who received liver transplantation remained free from disease at prolonged follow-up, thus the remaining 54% patients required palliative 5-FU based chemotherapy upon relapse. Authors did not specify whether patients after liver transplantation required “de novo” assessment of DPD activity compared to their initial pre-transplant assessment. Apparently, the risk of chemotherapy-related side effects in patients who received liver transplantation and chemotherapy opposed to patients who continued chemotherapy were not significantly different, albeit authors did not report the safety profile of chemotherapy just after liver transplantation, reporting the safety profile as a whole, thus including also the toxicity that was recorded in the pre-transplant phase.

The two patients that we have reported had received liver transplantation in different clinical settings but were found to have incidental discovery of intrahepatic cholangiocarcinoma (iCCA). To our knowledge, there are no published series reporting the administration of postoperative chemotherapy in iCCA after LT.

As liver transplantation surgery is not currently considered a standard of care and given that our patients have not undergone transplantation in clinical trials, we have deemed transplant surgery to be equivalent to liver resection.

In the cases reported, we recommended adjuvant treatment with Capecitabine monotherapy (1250 mg/sqm twice daily dd1-14 once every 3 weeks for 8 cycles), according to the BILCAP [24] schedule. As we aimed to reduce the risk of side effects from 5FU-based chemotherapy, DPD activity assessment was required before treatment start.

Both patients underwent *DPYD* polymorphisms analysis on DNA derived from a histological sample of the donor’s liver: plasma uracil concentrations as an alternative way to assess DPD activity were deemed to be inaccurate as they would be influenced by pre-existing hampered renal function.

Indeed, both patients had received liver transplantation in the management of liver cirrhosis and in one patient this was complicated by hepatorenal syndrome that was managed by heterotopic kidney transplantation: creatinine clearance of both patients was altered at baseline but eGFR was in the range as to allow start of Capecitabine based adjuvant chemotherapy. Because of this fact, *DPYD* polymorphisms assessment in donor’s liver tissue was the only reliable option for DPD activity assessment.

The time required for *DPYD* polymorphism reporting was 16 days in patient A and 21 days in patient B. Although it required longer times compared to standard *DPYD* polymorphism analysis (usually around 3 work-days), the time lag was due to the issue of tracing back the histologic specimen of the donor. Time required to have the result of DPD activity assessment did not allow us to start adjuvant treatment within the 8 weeks time-frame since initial surgery. This could be a major issue as it has been previously documented in several tumor types that in treatment beginning within 8 weeks should be preferred, in order to increase the likelihood to benefit from such treatment.

It could be safely assumed that the test would have quicker response times should the histologic specimen of the donor be easily available: recent trials of liver transplantation for liver metastases from colorectal cancer such as COLT trial [39] have already managed this issue by means of donor’s liver tissue storage, albeit in an ancillary sub-study. Based on this fact, we suggest that proper handling and storage of the pre-transplant liver biopsy should be done. This would allow to minimize the time required to have the result of this analysis as most of the time lag of the procedure was due to handling and shipping of the histologic specimen, rather than the test itself.

Both assessment resulted to be wild-type and allowed start of Capecitabine-based chemotherapy at full-dose (1250 mg/sqm twice daily for 14 days out of a 21 days period).

Both patients were able to complete 8 cycles of post-operative chemotherapy, albeit dose reduction were required: In patient A, due to increase of creatinine and worsening of renal function, dose of Capecitabine had to be reduced from 1250 mg/sqm bid to 1000 mg/sqm bid. The worsening of renal function was attributed to the fact that the patient had also received heterotopic liver transplantation for hepatorenal syndrome, rather than Capecitabine based toxicity. Indeed, eGFR remained stable throughout the rest of treatment and no severe side effects were recorded. In patient B, due to decrease in platelet count, dose reduction and dose delays were required: it must be noticed however that this patient started treatment with low platelet count due to splenomegaly and thus the impact of Capecitabine on platelet count was marginal.

There is limited evidence on the effectiveness of fluoropyrimidine treatment after LT, with the largest examples coming from studies on adjuvant chemotherapy after liver transplant for hepatocellular carcinoma and unresectable liver metastases of colon cancer [37, 41, 42]. However, none of these studies mention the method used to assess *DPYD* polymorphisms. Therefore, there are no evidence-based recommendations for managing patients with this complex presentation.

In the medical literature, there is only one other published case that tried to assess DPD deficiency in patient that required 5-FU based chemotherapy after LT [43]. This case involved a patient who had undergone LT to treat end-stage liver disease caused by primary sclerosing cholangitis.

The patient was later diagnosed with pancreatic cancer and needed fluoropyrimidine-based chemotherapy. However, the patient’s liver donor genotype was unknown and testing the DPD activity in peripheral blood cells was not indicative of liver activity due to different genotypes; the medical team proposed the use of therapeutic drug monitoring to ensure the patient’s safety.

During the first cycle of chemotherapy, the researchers reduced the initial dose of the 5-FU components by 30% and monitored the patient’s plasma concentration of 5-FU. Based on this result and the patient’s clinical observations, they were reassured that the patient did not have a severe DPD deficiency.

In this case, authors opted for monitoring 5-FU plasma concentrations as means to reduce the risk of toxicity instead of performing donor liver DPYD genotype as in our two patients. We believe that, owing to the increased number of patients who would be candidate to liver transplantation and who would require post-transplant 5-FU based chemotherapy, assessment based on 5-FU plasma concentration would not be feasible and sustainable: due to changes in patient’s liver conditions between different cycles of chemotherapy, drug monitoring would have to be done for each cycle of chemotherapy instead of just performing transplanted liver tissue *DPYD* genotyping only once before treatment. An increased number of patients would require a lot of resources just for this assessment and that would not be feasible to do in routine clinical practice.

In addition to that, the patient that was reported did receive polychemotherapy with FOLFIRINOX regimen: there are two additional limitations to drug monitoring as means to prevent treatment related side-effects that would be overcome by liver genotyping:

First of all, patient treatment schedule (once every 2 weeks) allowed for a reliable form of assessment of 5-FU circulating doses in the immediate time following chemotherapy infusion. On the other hand our patient received Capecitabine monotherapy continuously for 2 weeks out of a 3 weeks period: the prolonged exposure to Capecitabine would lead to some difficulties in interpreting the results of one single assessment of circulating 5-FU doses, thus making this form of testing as means of prediction of 5-FU toxicity less relevant.

Secondly, the patient received chemotherapy with FOLFIRINOX regimen: this chemotherapy combination includes, in addition to infusional 5-FU, also Oxaliplatin and Irinotecan. While Oxaliplatin metabolites are mainly excreted by kidneys and there is currently no evidence that liver cytochrome P-450 mediated metabolism is required [44], Irinotecan is extensively metabolized in the liver by UDP-glucuronosyltransferases (UGT) mediated processes.

Altered *UGT* gene polymorphisms have been also described to be associated with higher risk of Irinotecan-related treatment toxicity [45].

In order to fully estabilish safety profile of treatment in a liver transplant patient who is candidate to receive both 5-FU and Irinotecan combined, liver genotyping of both *DPYD* and *UGT* polymorphisms, by using the method that we described, would be able to manage this issue. On the other hand, by increasing the number of drugs that are used in the combination, drug monitoring would become increasingly more difficult and unfeasible.

## Conclusions

Patients who are treated with 5-FU based chemotherapy and who have received liver transplantation have rarely been described in the past and because of that there is a lack of knowledge concerning their optimal management. As *DPYD* polymorphisms analysis performed in the circulating blood cells is a mainstay before treatment start and because liver transplantation would nullify the usefulness of this testing due to different genotype between transplant receiver and donor, we suggest that alternative testing of *DPYD* polymorphisms on donor’s liver tissue should be performed. We have reported the cases of 2 patients where *DPYD* polymorphisms analysis was done on donor’s liver tissue to safely complete the scheduled adjuvant treatment for both patients. Due to the increase in applications of liver transplantation in patients who would also be candidate to receive 5-FU based chemotherapy, such as in colorectal cancer and cholangiocarcinoma, we also suggest that donor’s liver tissue should be routinely collected to perform this kind of testing.

## Electronic supplementary material

Below is the link to the electronic supplementary material.

Supplementary Material 1

## Data Availability

No datasets were generated or analysed during the current study.
