# Supplementary material for: Adjuvant treatment with Capecitabine in patients who received orthotopic liver transplantation with incidental diagnosis of intrahepatic cholangiocarcinoma. Implications on DPYD polymorphisms assessment: report of two cases and review of the literature
Source: Cancer Chemother Pharmacol. 2025 Mar 12;95(1):40. doi: 10.1007/s00280-025-04756-x (PMC11903612; doi:10.1007/s00280-025-04756-x)

**SUPPLEMENTARY FIGURES:**

Figure 1: PATIENT A images. 1A: Pre-OLT CT scan Post-Contrast Arterial Phase. 1B. Pre-OLT CT scan Post-Contrast Venous Phase.


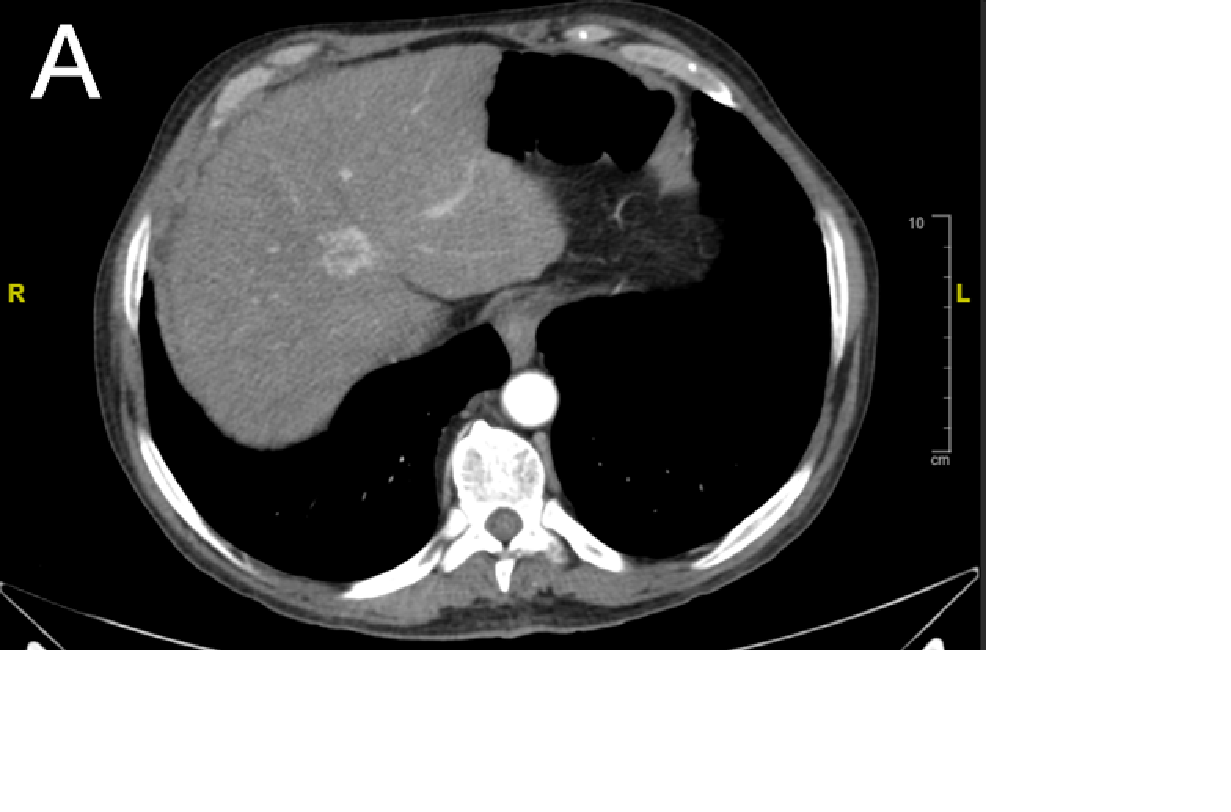

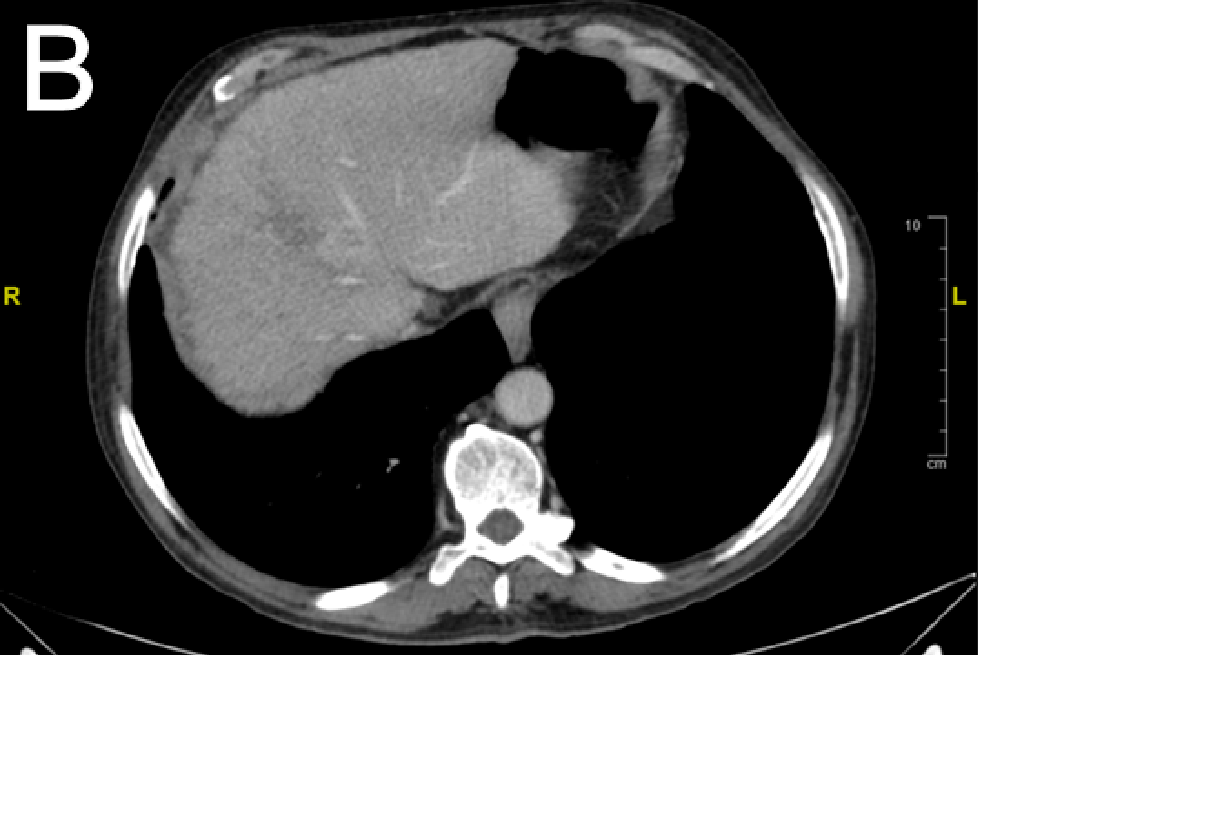


Figure 2. PATIENT B. images. 2A: Liver MRI at diagnosis. Nodules can be seen throughout the entire liver.


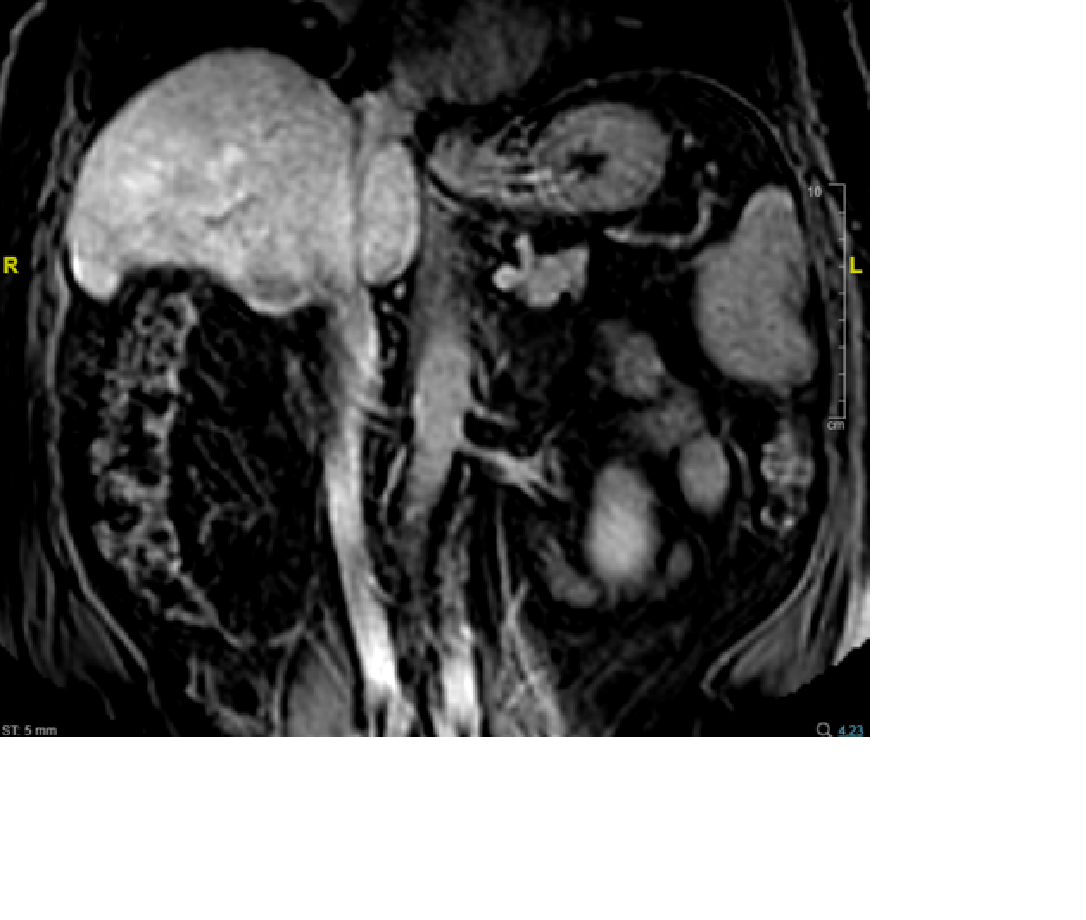

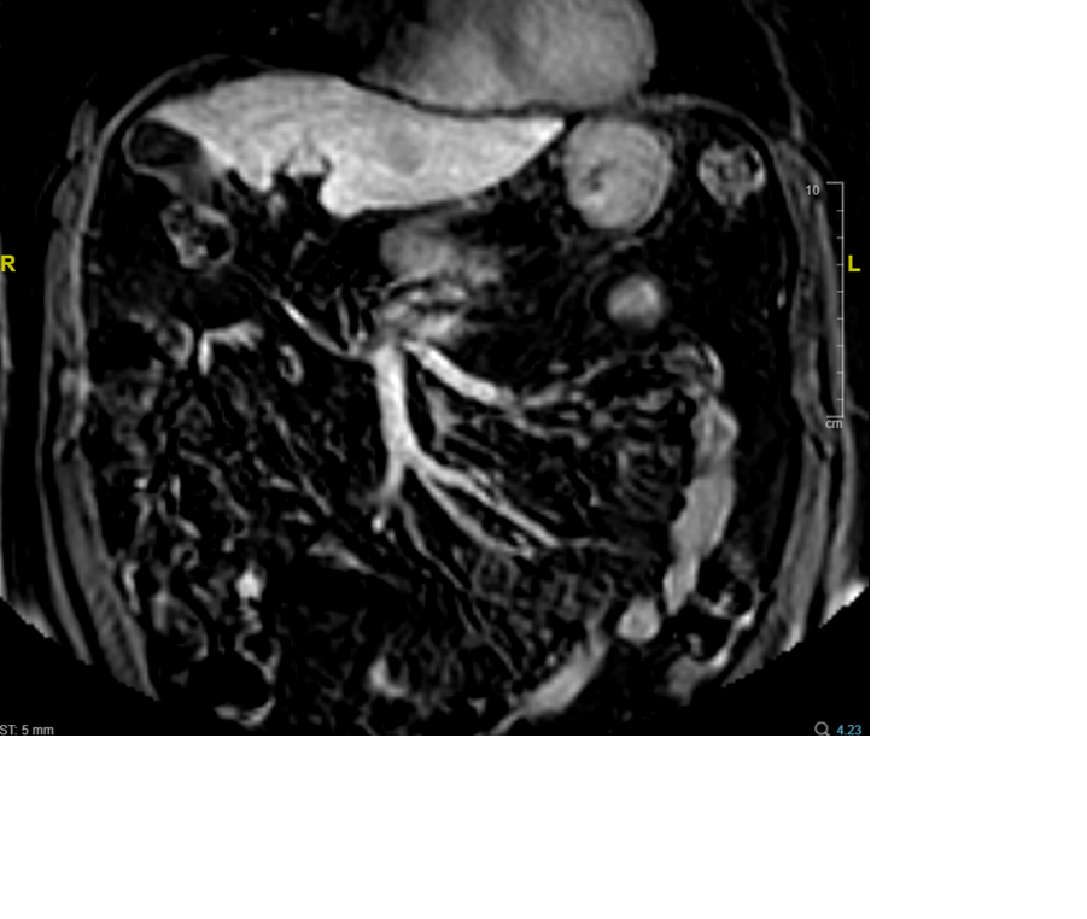

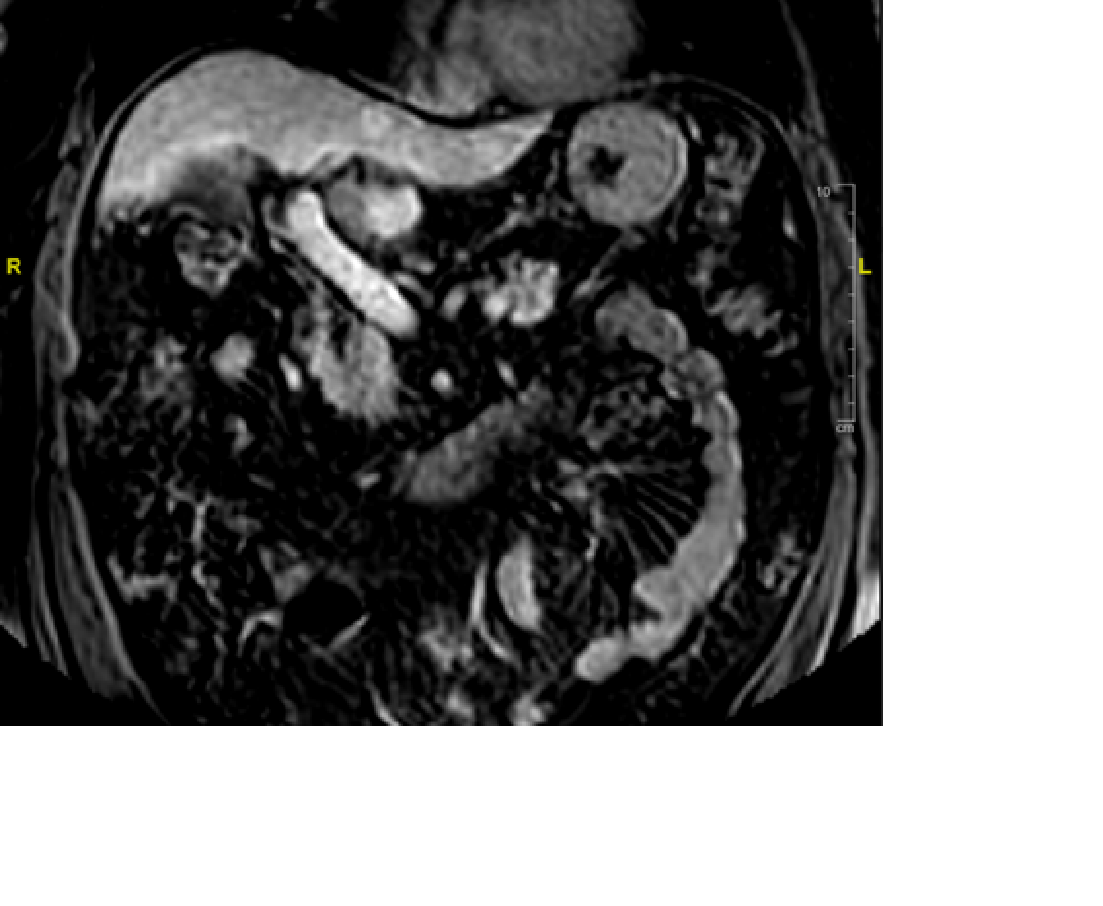

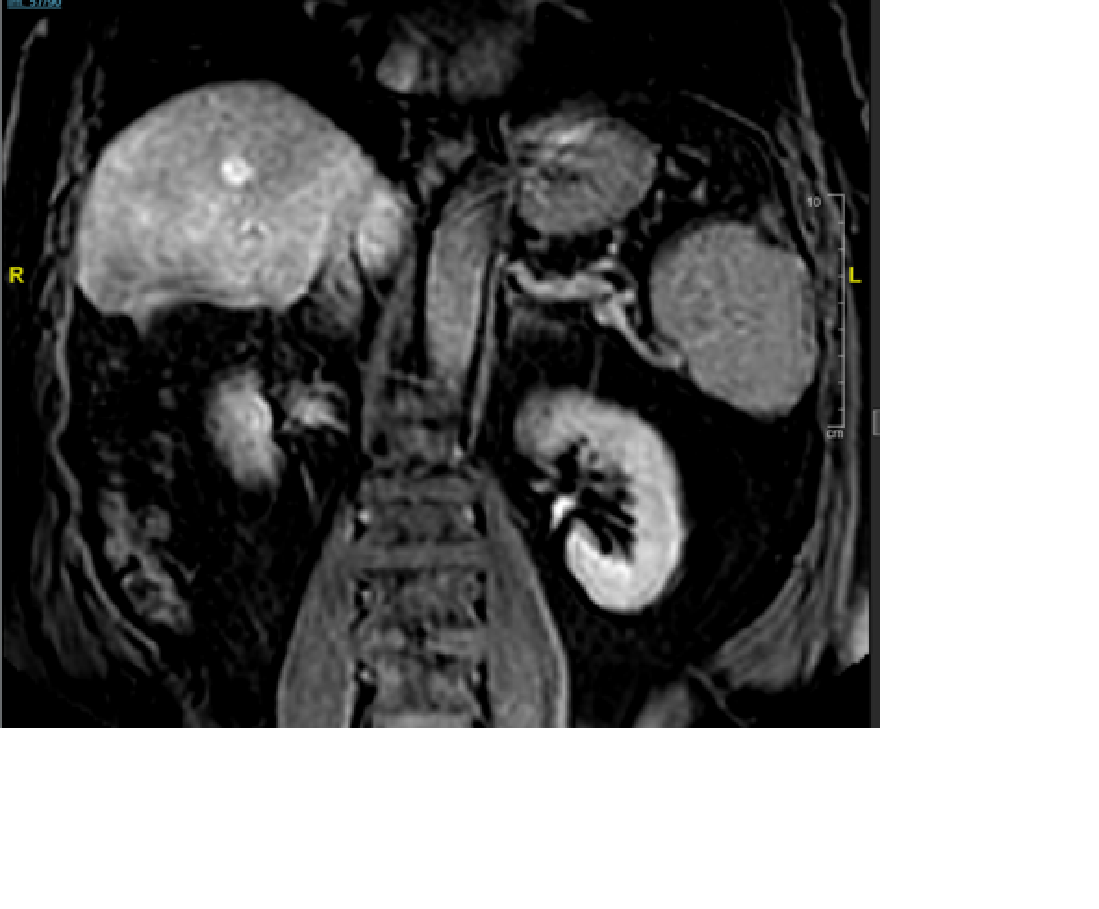


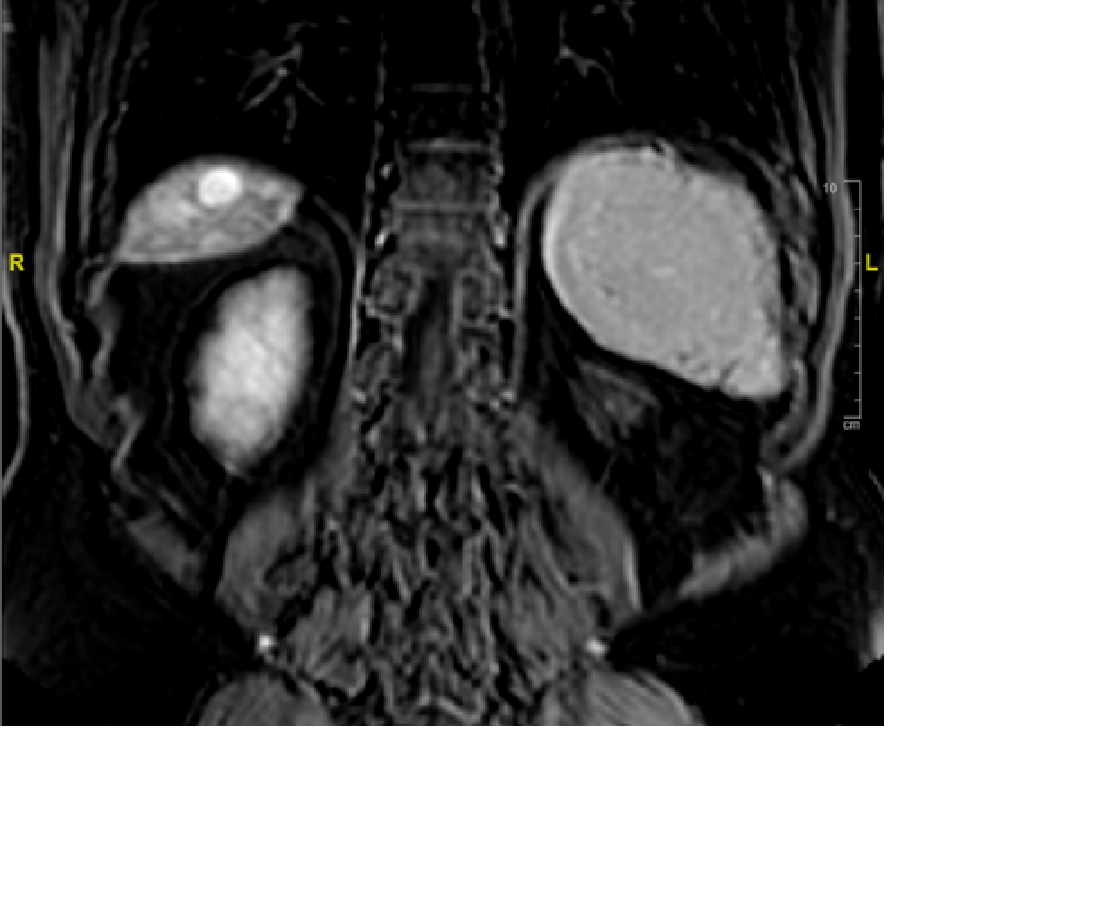


2B: Liver MRI after first TACE on 2 nodules on II liver segment.


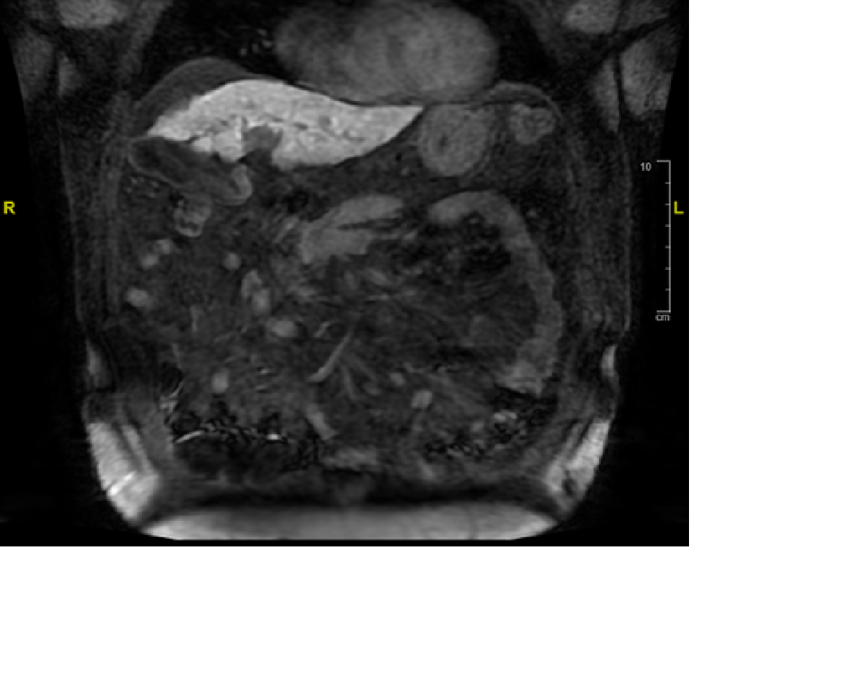

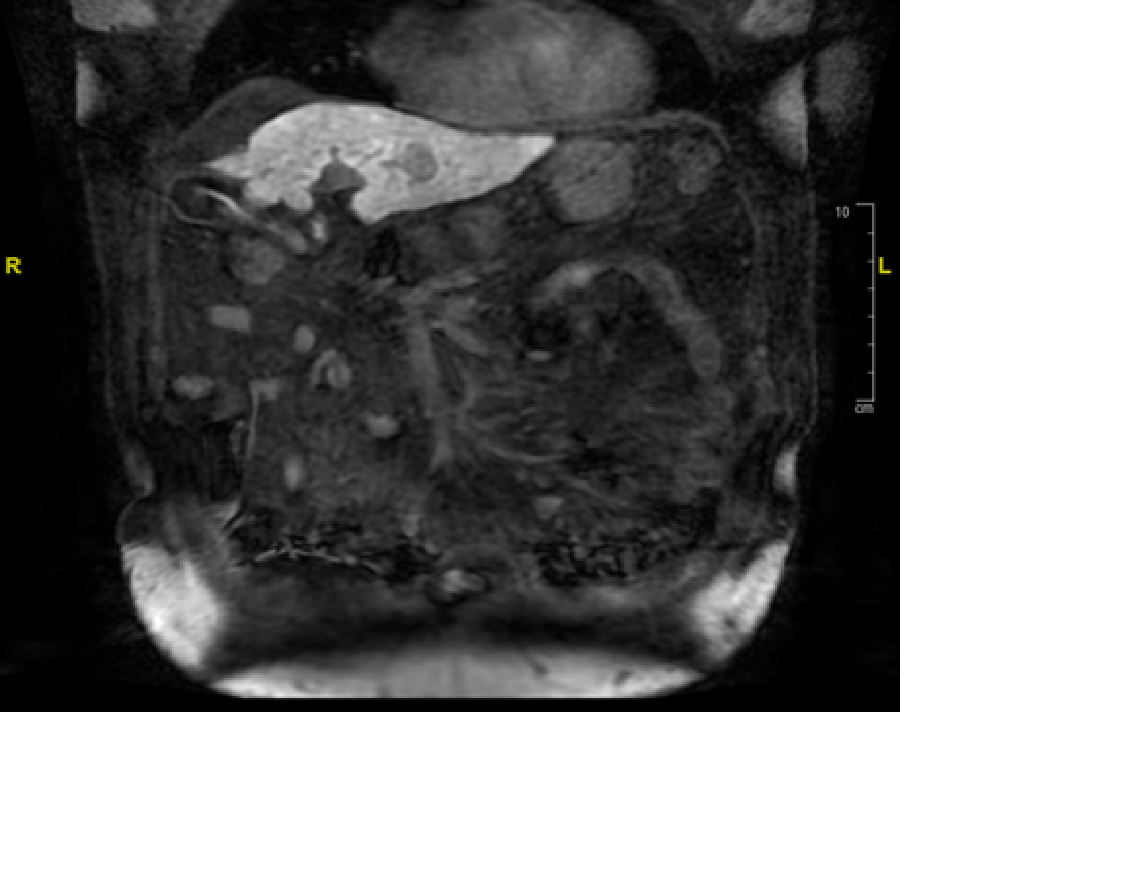


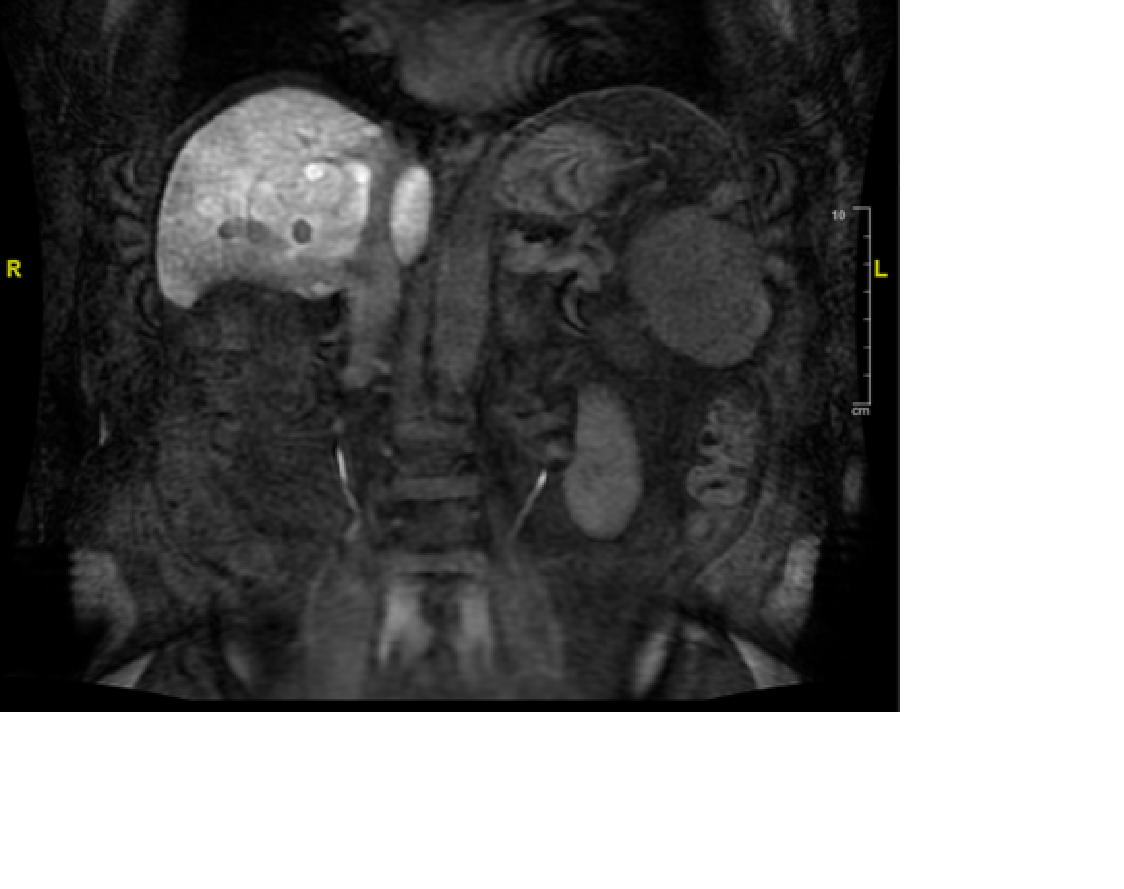

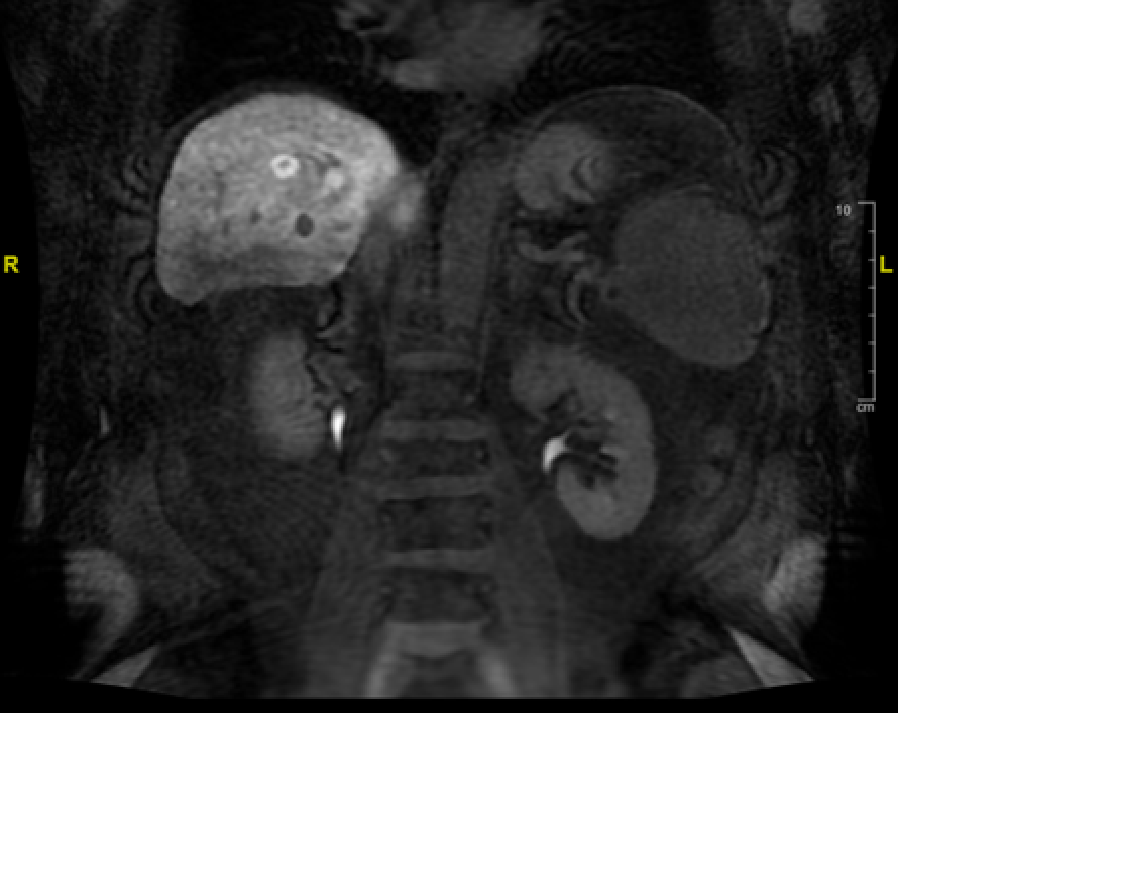


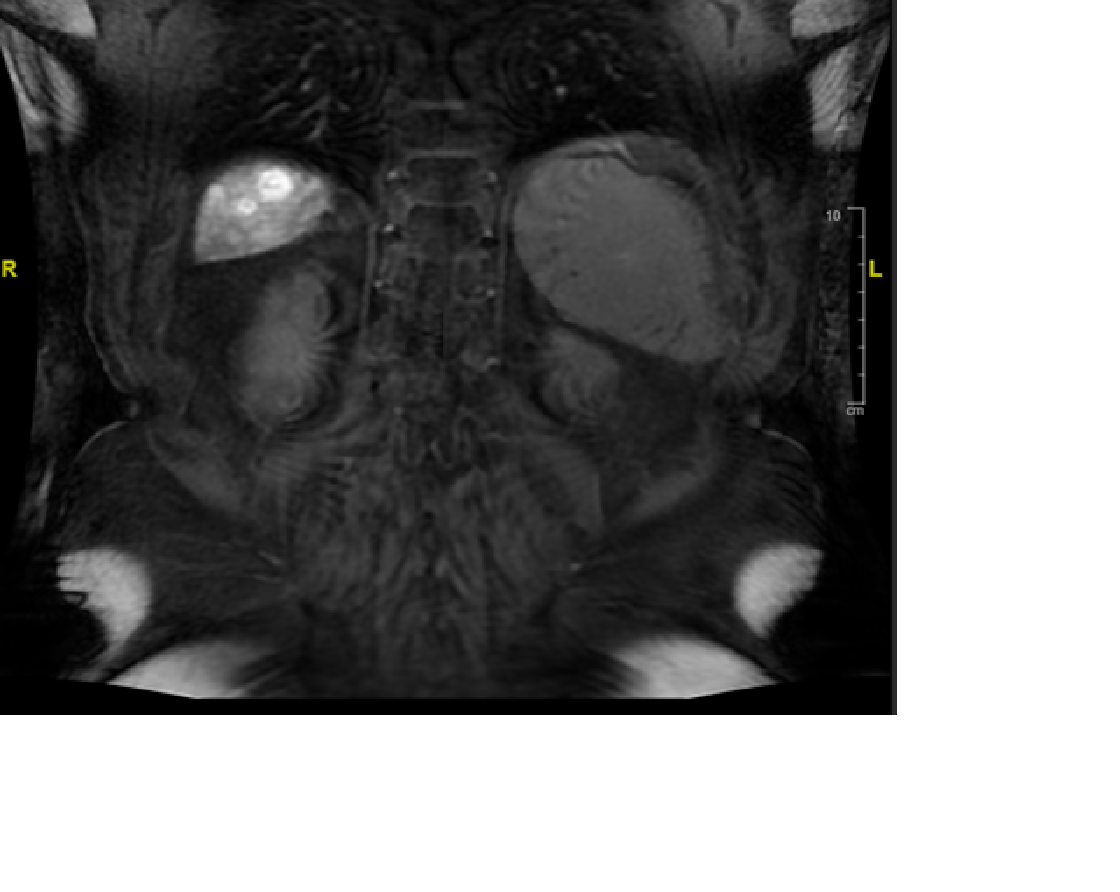


Figure 2C: MRI showing newly occurring nodule in S4.


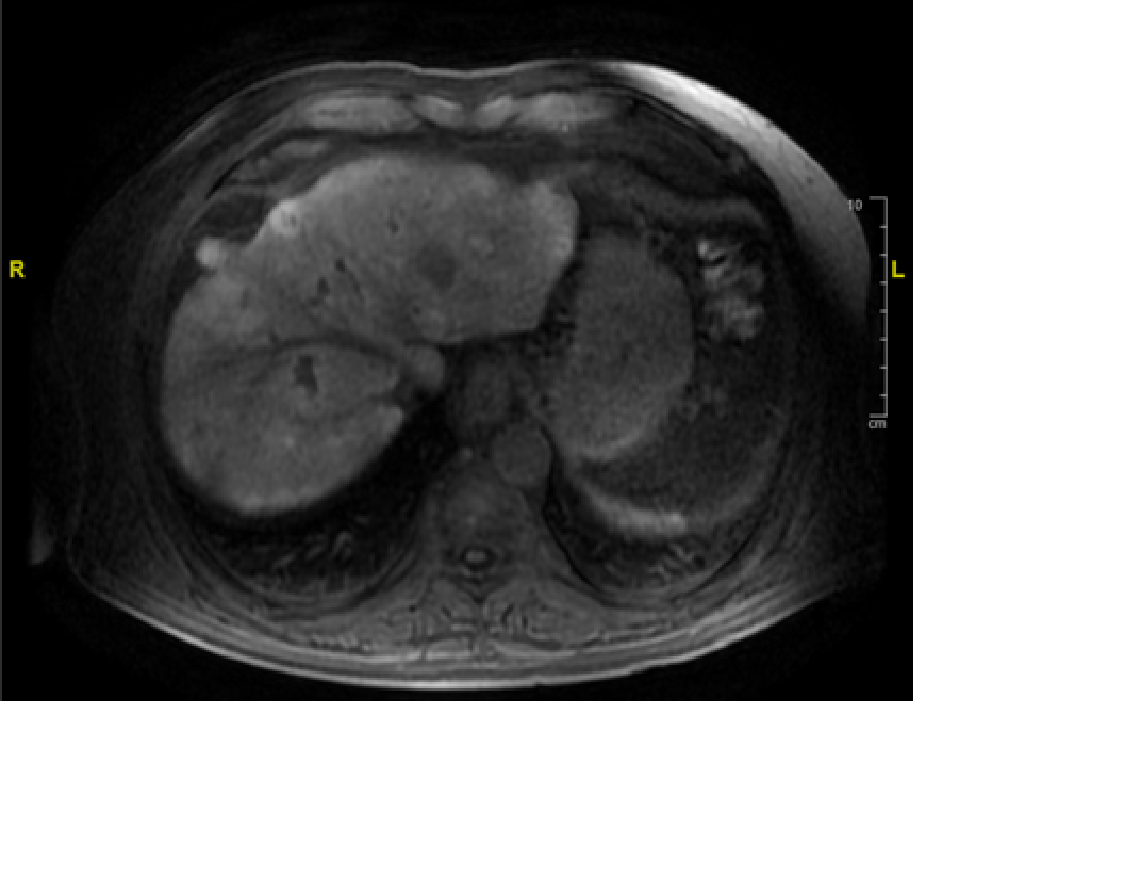


Figure 2D: MRI showing lack of response to 2^nd^ TACE.


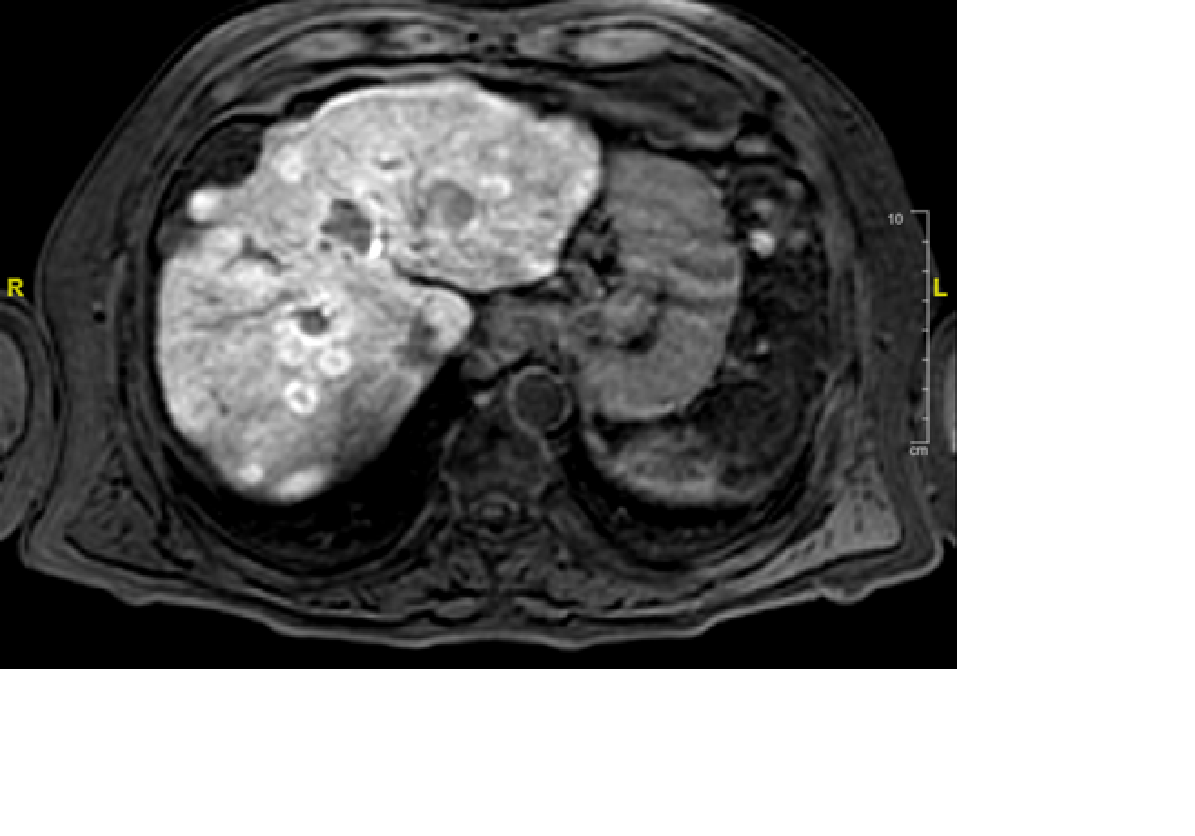

Supplement: Supplementary file 1 — Supplementary Material 1 [file 280_2025_4756_MOESM1_ESM.docx]
